# Mercury(II) Removal with Modified Magnetic Chitosan Adsorbents

**DOI:** 10.3390/molecules18066193

**Published:** 2013-05-24

**Authors:** George Z. Kyzas, Eleni A. Deliyanni

**Affiliations:** 1 Department of Chemistry, Aristotle University of Thessaloniki, Thessaloniki GR-541 24, Greece; E-Mail: lenadj@chem.auth.gr; 2 Department of Petroleum and Natural Gas Technology, Technological Educational Institute of Kavala, Kavala GR-654 04, Greece

**Keywords:** magnetic chitosan, cross-linking, mercury(II), adsorption

## Abstract

Two modified chitosan derivatives were prepared in order to compare their adsorption properties for Hg(II) removal from aqueous solutions. The one chitosan adsorbent (CS) is only cross–linked with glutaraldehyde, while the other (CSm), which is magnetic, is cross-linked with glutaraldehyde and functionalized with magnetic nanoparticles (Fe_3_O_4_). Many possible interactions between materials and Hg(II) were observed after adsorption and explained via characterization with various techniques (SEM/EDAX, FTIR, XRD, DTG, DTA, VSM, swelling tests). The adsorption evaluation was done studying various parameters as the effect of pH (optimum value 5 for adsorption and 2 for desorption), contact time (fitting to pseudo–first, –second order and Elovich equations), temperature (isotherms at 25, 45, 65 °C), in line with a brief thermodynamic analysis (ΔG^0^ < 0, ΔH^0^ > 0, ΔS^0^ > 0). The maximum adsorption capacity (fitting with Langmuir and Freundlich model) of CS and CSm at 25 °C was 145 and 152 mg/g, respectively. The reuse ability of the adsorbents prepared was confirmed with sequential cycles of adsorption-desorption.

## 1. Introduction

Chitosan (poly–β–(1→4)–2–amino–2–deoxy–D–glucose) is an aminopolysaccharide and cationic polymer produced by the *N*–deacetylation of chitin. It is one of the most naturally abundant and cheap biopolymers. It is a hydrophilic, nontoxic, biodegradable, and biocompatible material with the ability to form complexes with metals [1,2,3]. The latter could be easily explained by the presence of amino groups on the polymer matrix, which can interact with metal ions of the solution by ion exchange or other complexation reactions (mainly chelation) [4,5]. The high content of amino groups also makes possible many chemical modifications on the polymer with the purpose of improving its adsorption features, such as selectivity and adsorption capacity [6,7]. Its adsorption performance can be further improved by cross-linking with reagents such as glutaraldehyde, tripolyphosphate salts, epichlorohydrin, ethylene glycol or diglycidyl ether, which can stabilize chitosan in acid solutions and increase its mechanical properties [8].

Chitosan has been repeatedly characterized as a metal superadsorbent [Cu(II), Cd(II), Pb(II), Ni(II), Hg(II), Cr(VI), U(VI), Mo(V), V(V), Pd(II), Pt(IV), Au(III), As(V), Se(V)] presenting significantly high adsorption capacities (0.2–8.0 mmol/g) [4,9]. Their maximum adsorption capacity is varied, which is attributed to: (i) the form of chitosan (beads, powder), (ii) the possible chemical modifications (cross-linking, grafting reactions), (iii) the different experimental conditions (pH, particle size, conditioning, and composition of the solution), which are not systematically the same [4]. The latter is the reason why the direct comparison of experimental data is not possible.

In the present study, Hg(II) was selected as target for removal with adsorption technique, which is considered to be one of the most effective and economical treatment methods for metal removal in the general field of separation processes of chemical technology [4,9,10]. In general, contamination of aquatic systems is a serious environmental problem given the pollution of natural waters by heavy metal ions is one of the main sources all over the World. Mercury is one of the most toxic heavy metals since it is not biodegradable and causes a lot of toxic effects in the human body [11]. Its presence is due to a combination of natural processes (volcanic action, erosion of mercury–containing sediments) and anthropogenic activities (mining operations, tanneries, metal plating facilities) as well [12]. Although numerous works have been published aiming the removal of various metals (or especially Hg(II) [13,14,15,16]) with adsorption onto chitosan materials [4,9], a great of importance is the modification of chitosan (apart from the common chitosan derivatives grafted with carboxyl, amino, amido, imino, sulfonate, *etc.* [17,18,19,20,21]) with magnetic nanoparticles in order to form a magnetic chitosan derivative. Some magnetically modified chitosans were already tested for the removal of many metals in literature [22,23,24,25]. However, there is lack of studied for the use of magnetic chitosan powders (not resins or membranes) as adsorbent for mercury(II) removal [26,27].

In the current study, a modification of pure chitosan was realized to form two differently modified derivatives: (i) cross–linked with glutaraldehyde (denoted as CS), and (ii) cross–linked with glutaraldehyde and functionalized with magnetic nanoparticles (denoted as CSm). Many possible interactions between materials and Hg(II) were observed after adsorption and explained via characterization with various techniques (SEM/EDAX, FTIR, XRD, DTG, DTA, VSM, swelling tests). The adsorption evaluation was done studying various parameters as the effect of pH, contact time, temperature, in line with a brief thermodynamic analysis. The reusability of the adsorbents prepared was confirmed with sequential cycles of adsorption–desorption, given the technological field of this study.

## 2. Results and Discussion

### 2.1. Characterization

**Figure 1:**
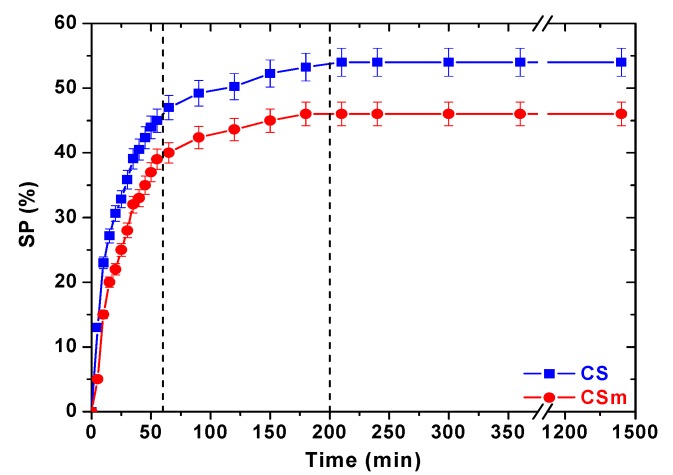
Effect of contact time on swelling percentage.

It is well–known that chitosan derivatives present high swelling percentages [28]. The swelling percentage was found to be 46% for CSm and a little bit higher (54%) for CS. Therefore, given the initial mass of adsorbent used was adjusted to 0.02 g, the calculated water content was 0.0092 g for CS and 0.0108 g for CSm. The slight increase can be due to the absence of functionalized magnetic nanoparticles on CSm, which gave a more compact structure on the magnetic derivative. In addition, the kinetics of swelling (Figure 1) revealed the similar behavior of two chitosans (CS and CSm). There is a steep ascent within 1 h being succeeded by a more gradual rise for both adsorbents. After 200 min, the swelling of water seemed to be completed (equilibrium stage).

**Figure 2:**
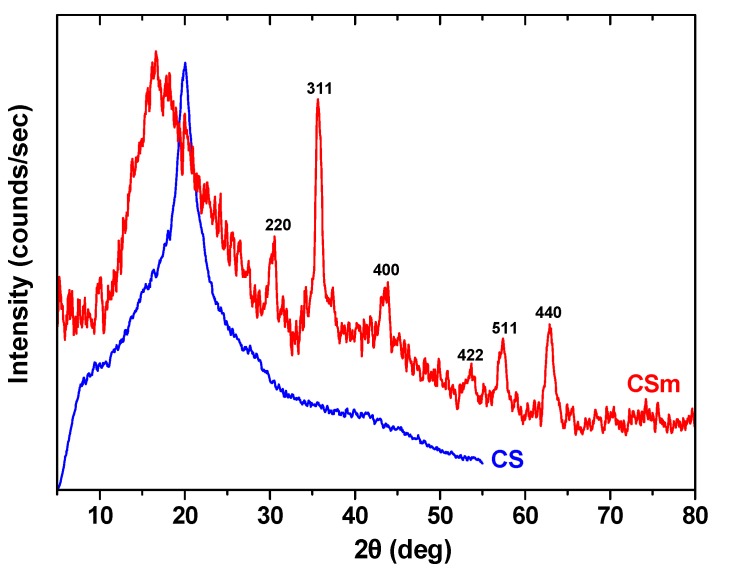
X–ray diffraction (XRD) patterns of CS and CSm.

The XRD patterns of CS and of CSm are shown in Figure 2. In the X–ray diffraction spectrogram of CS, a broad peak at 2θ = 20.2° is presented due to the amorphous state of chitosan. In the spectrogram for the magnetic sample (CSm), the six characteristic peaks of Fe_3_O_4_ at 2θ = 30.1°, 35.5°, 43.3°, 53.4°, 57.2°, and 62.5°, attributable to the indices (220), (311), (400), (422), (511), and (440), respectively, were observed. They were indexed using the Joint Committee on Power Diffraction Standards database (File No. 19–0629) to Fe_3_O_4_ with a cubic inverse spinel structure, indicating the presence of magnetite particles [29]. The results showed that chitosan binding did not result in a phase change in the structure of the magnetic nanoparticles. The average crystallite size, D (nm), of magnetite was calculated about 10.5 nm, using the Debye–Scherrer equation [30]:

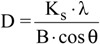
(1)
where K_s_ is a constant (K_s_ = 0.9 for CuKa), λ (nm) is wavelength (0.15405 nm for CuKa), B is the peak width of half–maximum (rad) and θ is the diffraction angle. The patterns of CSm also exhibited a broad peak at 2θ = 20.3° due to the presence of chitosan. 

**Figure 3:**
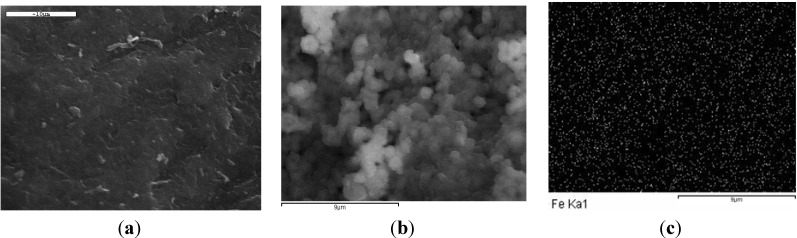
SEM images of: (**a**) CS; (**b**) CSm; (**c**) Fe distribution map of CSm.

SEM analysis provided information about the size and the surface morphology of magnetic chitosan. The SEM image of CSm (Figure 3) shows that it had a spherical shape and the final products presented aggregation. It is concluded that chitosan was immobilized on the surface of Fe_3_O_4_ particles with a core–shell construction of an average diameter about 1 μm. The map images (Figure 3b) present a confirmation of the success of the chitosan coating process on the surface of Fe_3_O_4_ particles with a good distribution. EDX analysis determined the chemical composition of the CSm. The elemental percentage of carbon was found to be 52.30%, while the respective of oxygen was 22.44% and 25.36% of iron.

**Figure 4:**
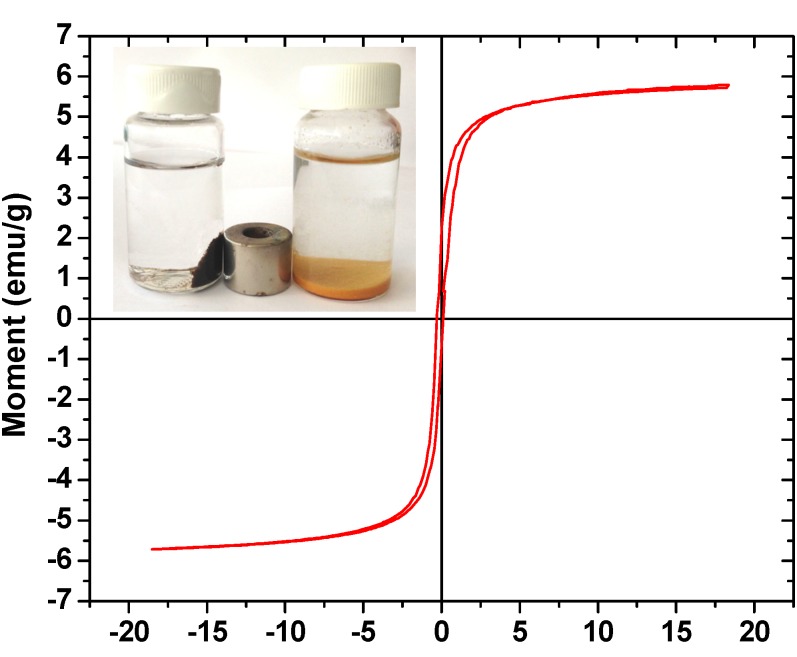
VSM plot of CSm (Inset: photo of CS and CSm after Hg(II) adsorption).

The magnetic property was measured on a vibrating sample magnetometer (VSM) and presented via VSM plot. According to Figure 4, the VSM plot showed a value of 5.79 emu/g for the saturation magnetization of CSm. This value is far less than that reported for pure magnetite colloidal nanocrystals (36.941 emu/g) [31], but it may be due to the rather small size and the relatively low amount of Fe_3_O_4_ loaded on CSm. In any case, the magnetic property remained high enough for a magnetic separation to be achieved. Because of the presence of magnetic particles, using an external magnetic field, CSm could be easily and rapidly separated from the aqueous solution after the experiments. In the inset of Figure 4, the right vial contains an aqueous solution of Hg(II) after adsorption onto CS, whereas the left vial contains an aqueous solution of Hg(II) after adsorption onto CSm. In the presence of an external magnetic field, the black particles of the CSm were attracted to the wall of the vial to emphasize the magnetism of the prepared CSm. So, a brief comparative table (Table 1) presents the differences of the main physical properties of chitosan adsorbents prepared. 

**Table 1:** Properties of the adsorbents.

Property Type	Adsorbent	
	CS	CSm
	powder	powder
SP (%)	54	46
Water content (g)	0.0092	0.0108
Particle size (μm)	75–125	75–125
Surface area (m^2^/g)	<3	<3
Porosity	non–porous	non–porous
Magnetization (emu/g)	–	5.79
Iron (%)	–	25.36

**Figure 5:**
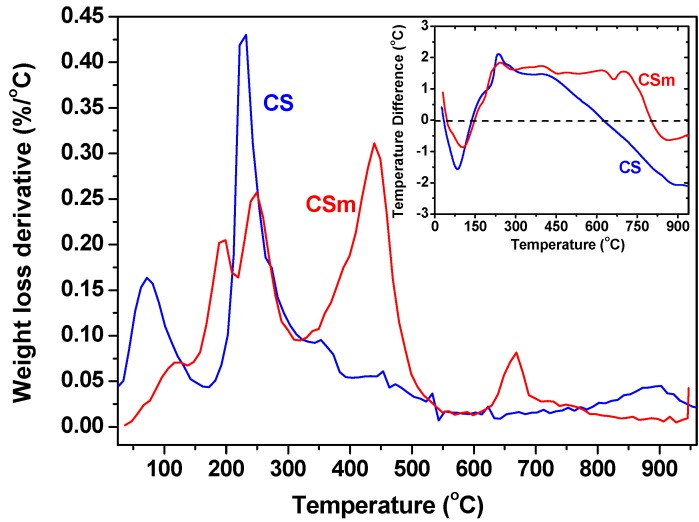
Differential thermogravimetry (DTG) curves in nitrogen (inset: differential thermal analysis (DTA) curves in nitrogen).

Differences in the chemistry of the surfaces are presented on differential thermal gravimetric (DTG) curves measured in nitrogen (Figure 5), where the peaks represent weight loss at the specific temperature range. The first peak centered at about 80–100 °C for CS and CSm, in correlation to the endothermic effect on the differential thermal analysis (DTA) curves presented in the inset of Figure 5, can be linked to weight loss due to the evaporation of physically adsorbed water. In the case of CS, that peak presented a maxima at 100 °C, while a second peak with a higher mass loss was found at 400 °C, caused by the decomposition of CS derivatives containing hydroxyl and amino groups [32]. CSm displayed a broad exothermic peak around 270 °C that can be attributed to the decomposition of chitosan while the weight loss in the range 450–500 °C should be assigned to the further decomposition of chitosan residues, whereas the band in the range of 600–900 °C can be attributed to the reduction of Fe_3_O_4_ by reaction with residual carbon [29]. 

**Figure 6:**
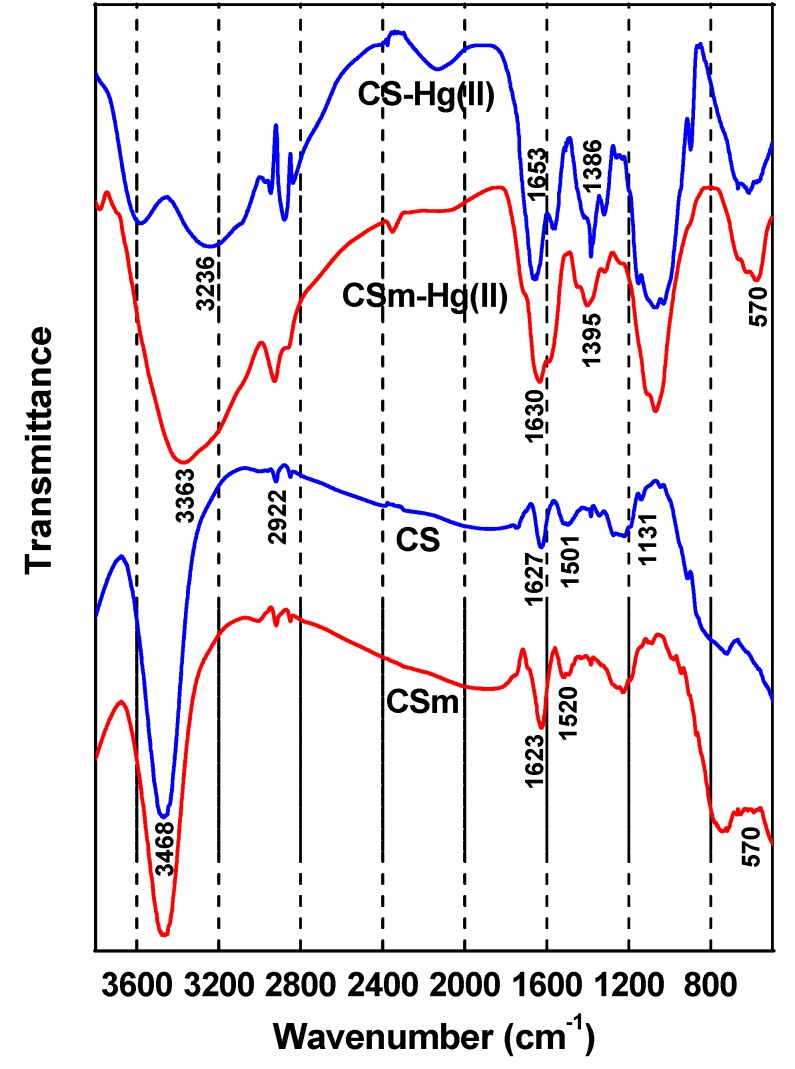
FTIR spectra of CS and CSm before and after Hg(II) adsorption.

FTIR spectra (Figure 6) revealed the differences in the chemical features of the raw materials, as well as for the materials after adsorption. In the spectrum of CS a strong peak around 3,400 cm^–1^ was appeared, due to the stretching vibration of O–H, the extension vibration of N–H and the inter-hydrogen bonds of the polysaccharide [33]. The C–H stretching vibration of the polymer backbone is manifested through strong peak at 2,922 cm^–1^ while the two characteristic bands centered at 1,627 and 1,501 cm^–1^ (1,520 cm^–1^ for CSm) can be attributed to the C = O stretching vibration mode of NHCO (amide I) along with an N–H deformation mode and the N–H bending of NH_2_ group, respectively [20]. The band at 1,217 cm^−1^ can be attributed to C–N stretching vibration (amino group band) and the band at 1,096 cm^−1^ to the stretching vibration mode of the hydroxyl group. Characteristic bands of chitosan appeared at 1,131 cm^−1^ due to the special broad peak of (1–4)–glucosidic band in polysaccharide unit, at 1,028 cm^−1^ due to the stretching vibration of C−O−C in the glucose circle, and at the 1,060−1,015 cm^−1^ corresponding to CH–OH in cyclic compounds [20]. In the spectrum of CSm, in addition to the characteristic absorption bands of the functional groups of chitosan, a new peak appeared at 570 cm^−^^1^, related to Fe–O group, demonstrating that CS was coated successfully to the magnetic Fe_3_O_4_ nanoparticles via electrostatic interaction, results consistent to the core–shell SEM images. The surface of iron oxide possessed negative charges having this way an affinity toward CS, so, the protonated CS could coat the magnetite nanoparticles by the electrostatic interaction and chemical reaction through glutaraldehyde cross–linking [34,35]. 

**Figure 7:**
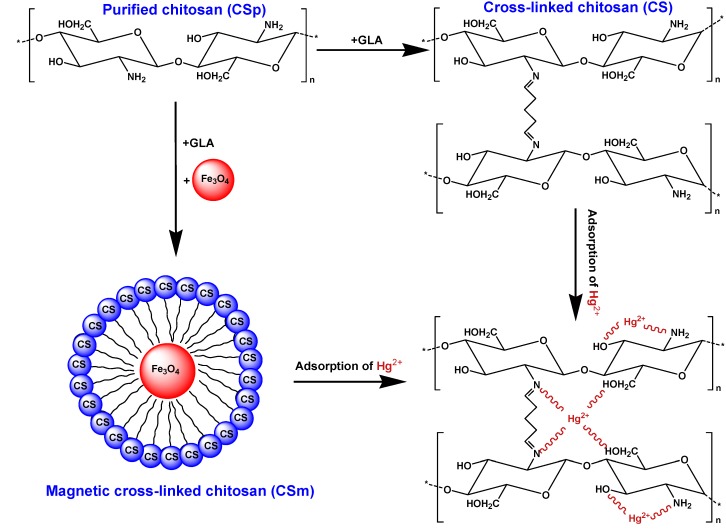
Preparation of CS and CSm and possible interaction with Hg(II).

In the spectra of CS–Hg(II) and CSm–Hg(II), the interactions were mainly affirmed by the reduction of the intensity of the peak assigned to the –NH group in amine, confirming that nitrogen atoms are the main adsorption sites for Hg(II) adsorption on CS and CSm. It is well known that nitrogen–ligand possesses affinity to adsorb Hg(II), which belongs to soft acid [36] leading to the mercury chelation with nitrogen atoms of amino groups [37,38,39,40]. On the other hand, the peaks at 1627 and 1,623 cm^−^^1^ for CS and CSm, characteristic peak of –NHCO, shift to 1,653 and 1,630 cm^−^^1^ respectively, evidence of the coordination of –NHCO with Hg(II). It is also observed that, after Hg(II) sorption, appeared a new band at 1,386 cm^−^^1^ [CS–Hg(II)] and 1,395 cm^−^^1^ [CSm–Hg(II)], attributed to C–N stretching vibration [40]. The presence of this peak indicated that even after cross-linking, the structure resulting from the primary amino and glutaraldehyde reaction (imine bond) is also capable of adsorbing metallic cations [41]. This may be attributed to the presence of free lone pair of electrons on nitrogen atoms at pH 5.0, suitable for coordination with the metal ion to give the corresponding magnetic chitosan–metal complex [42]. In addition, the peak at 3,468 cm^−^^1^ corresponding to the stretching vibration of OH–groups shifts to 3,236 cm^−^^1^ [CS–Hg(II)] and 3,363 cm^−^^1^ [CSm–Hg(II)] after Hg(II) sorption, indicating that –OH groups also take part in sorption. This conclusion was consistent with other studies for other metel ions. Li and Bai [43] showed that the binding of lead ions to nitrogen atom in chitosan and oxygen atom in –OH group may contribute to lead adsorption on chitosan/PVA beads. Other studies also found that –OH and –NH_2_ was involved in adsorption process [40,44]. Furthermore, despite the bonds of GLA with amino groups of chitosan (after the formation of CS or CSm), there are still free amino groups able to interact (proposed chelation reaction of Figure 7). The proposed chelated structures of Hg(II) with CS and CSm is presented in Figure 7.

### 2.2. Adsorption

#### 2.2.1. Effect of pH

**Figure 8:**
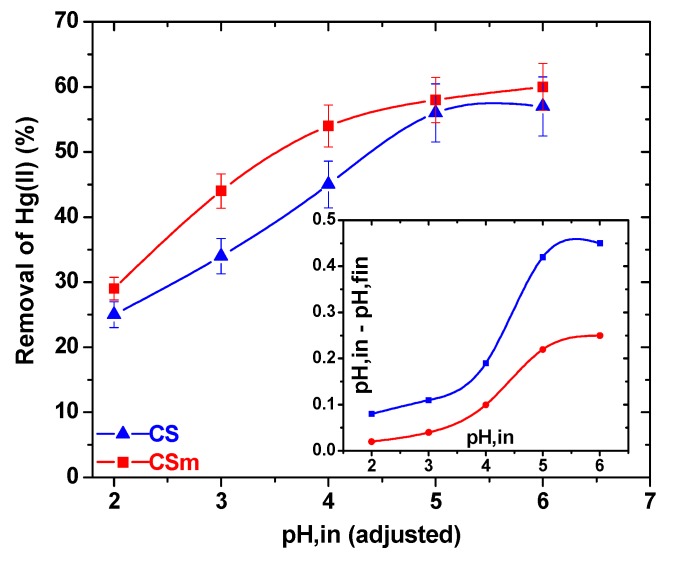
Effect of pH on adsorption (Inset: Change of pH between initial/adjusted conditions and final).

The effect of pH was revealed a similar behavior for CS and CSm. As presented in Figure 8, at pH = 2 both adsorbents presented their lowest Hg(II) uptake (25% and 29% for CS and CSm, respectively), while at pH = 3 the respective values were 34% and 44%, so a change of 9% and 15% was observed. Reaching in less acidic pH conditions (pH = 4), the metal uptake was further increased to 45% and 54% for for CS and CSm, respectively. At pH = 5 and pH = 6, the metal uptakes were stabilized to 56% and 57% for CS and 58% and 60% for CSm. Higher pH values were not studied in order to avoid precipitation. At a first glance, both adsorbents presented similar trend in their metal uptake increase, but in any case CSm showed a slight superiority in metal uptake than CS. According to the above experimental findings, the optimum pH found was 5 for both materials. This may be attributed to the presence of free lone pair of electrons on nitrogen atoms of chitosan suitable for coordination with the metal ion to give the corresponding chitosan–metal complex (Figure 7). It is selected to avoid the value of pH = 6, which is near to the crucial zone of precipitation, where the metal ions get out of the solution due to formation of colloidal precipitate of Hg(OH)_2_ and not due to the adsorption of free Hg(II) ions [26]. In general the uptake of Hg(II) at pH > 6 is attributed to the formation of metal hydroxide species such as soluble Hg(OH)^+^ and/or insoluble precipitate of Hg(OH)_2_. Furthermore, a brief comment can be done about the change of values between the initial (adjusted) pH of solution and the final [after Hg(II) adsorption] (inset of Figure 8). At the optimum/studied pH value (5), the pH change was 0.42 for CS and 0.21 for CSm. So, the slight change can be easily ignored. The latter was a strong reason for not adjusting the pH value during adsorption (continuously), preferring a free–pH process. In this way, the adsorption did not direct to fixed adsorption paths (with possible additions of H^+^ or OH^–^ in the case of continuous pH–adjustment), but govern only by the interactions with Hg(II).

#### 2.2.2. Effect of Contact Time

**Figure 9:**
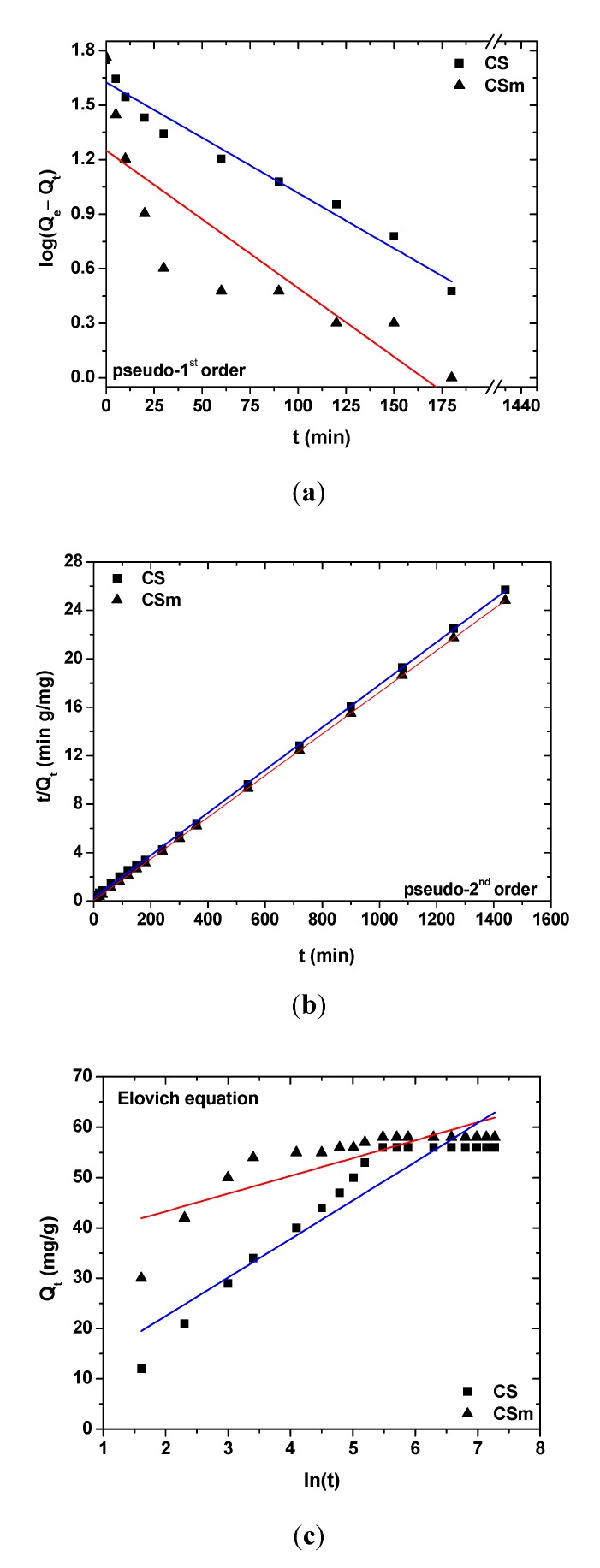
Effect of contact time on adsorption of Hg(II) onto CS and CSm: fitting to (**a**) pseudo–first order equation; (**b**) pseudo–second order equation; (**c**) Elovich equation.

Figure 9a,b,c show the effect of contact time on adsorption. The fitting was performed using pseudo–first, –second order and Elovich equations. Table 2 presents the kinetic parameters resulted from the fitting. Figure 9a shows the plot of linearization of pseudo–first order model [45], where the slope (–k_1_/2.303) and intercept log(Q_e_) of plot log(Q_e_–Q_t_) *versus* t was used to determine the pseudo-first order constant k_1_ and the equilibrium adsorption density Q_e,cal_. However, the experimental data deviated considerably from the theoretical data. The correlation coefficients (R^2^) obtained were not as high as those for pseudo–second equation (R^2^_CS_ = 0.964 and R^2^_CSm_ = 0.729). Also, the adsorption equilibrium values (Q_e,cal_) found gave significant deviation for both adsorbents. In the case of CS, the difference was 14 mg/g (Q_e,cal_ = 42 mg/g and Q_e,exp_ = 56 mg/g), while for CSm the deviation was very high, equal to 40 mg/g (Q_e,cal_ = 18 mg/g; Q_e,exp_ = 58 mg/g). These findings suggest that this adsorption system is not a pseudo–first order reaction. 

**Table 2:** Kinetic constants for the adsorption of Hg(II) onto CS and CSm ([Hg(II)]_0_ = 100 mg/L).

		Pseudo–first order			Pseudo–second order			Elovich equation		
	Q_e,exp_	k_1_	Q_e,cal_	R^2^	k_2_	Q_e,cal_	R^2^	a	β	R^2^
**Adsorbent**	(mg/g)	(min^–1^)	(mg/g)		(g mg^–1^ min^–1^)	(mg/g)		(mg g^–1^ min^–1^)	(g/mg)	
CS	56	0.0138	42	0.964	11.68 × 10^–4^	56	0.999	39.33	0.0276	0.658
CSm	58	0.0174	18	0.729	57.65 × 10^–4^	58	0.999	20.87	0.1395	0.884

Furthermore, the experimental data fitted to the pseudo–second order equation (Figure 9b), calculating the respective parameters [46]. The slope (1/Q_e_) and intercept (1/k_2_Q_e_^2^) of plot (t/Q_t_) *versus* t were used to calculate the parameters of k_2_ and Q_e,cal_. The straight lines in plots of Figure 9(b) showed an excellent agreement of experimental data with this model. The correlation coefficients for all adsorbents were equal to 0.999. Also, the calculated Q_e,cal_ values are completely the same with those exported from the experimental data (CS: Q_e,cal_ = Q_e,exp_ = 56 mg/g; CSm: Q_e,cal_ = Q_e,exp_ = 58 mg/g). These findings indicate that the adsorption system studied belongs to the second–order kinetic model. A comment can be given for the adsorption rate, comparing the kinetics constants (k_2_). According to Table 2, a relationship of k_2,CSm_/k_2,CS_ = 4.67 (k_2,CSm_ = 57.65×10^–4^ g mg^–1^ min^–1^ and k_2,CS_ = 11.68 × 10^–4^ g mg^–1^ min^–1^) can be easily extracted, revealing that magnetic chitosan adsorb/interact faster with Hg(II) ions than the non–magnetic derivative (CS). The latter can be attributed to the existence of Fe_3_O_4_ on CSm backbone, which may attract the ions.

Figure 9(c) shows a plot of linearization of Elovich model [47]. The slope and intercept of plots of Q_t_ *versus* ln(t) were used to determine the constant β_el_ and the initial adsorption rate α. However, the experimental data deviated considerably from the theoretical data. A comparison of the results with the correlation coefficients is shown in Table 2. The correlation coefficients for the Elovich kinetic model obtained at all the studies concentrations were low (R^2^_CS_ = 0.658 and R^2^_CSm_ = 0.884). This suggests that this adsorption system is not an acceptable for this system.

#### 2.2.3. Isotherms-Thermodynamics

The experimental data were fitted to the Langmuir [48], and Freundlich [49] isotherm model. Although the Langmuir and Freundlich isotherms were firstly introduced about 90 years ago, they still remain the two most commonly used adsorption isotherm equations. Their success undoubtedly reflects their ability to fit a wide variety of sorption data quite well. The Langmuir model represents chemisorption on a set of well defined localized adsorption sites, having the same adsorption energies independent of surface coverage and no interaction between adsorbed molecules. Langmuir isotherm assumes monolayer coverage of adsorbate onto adsorbent. Freundlich isotherm gives an expression encompassing the surface heterogeneity and the exponential distribution of active sites and their energies. This isotherm does not predict any saturation of the adsorbent surface; thus, infinite surface coverage is predicted, indicating physisorption on the surface.

**Figure 10:**
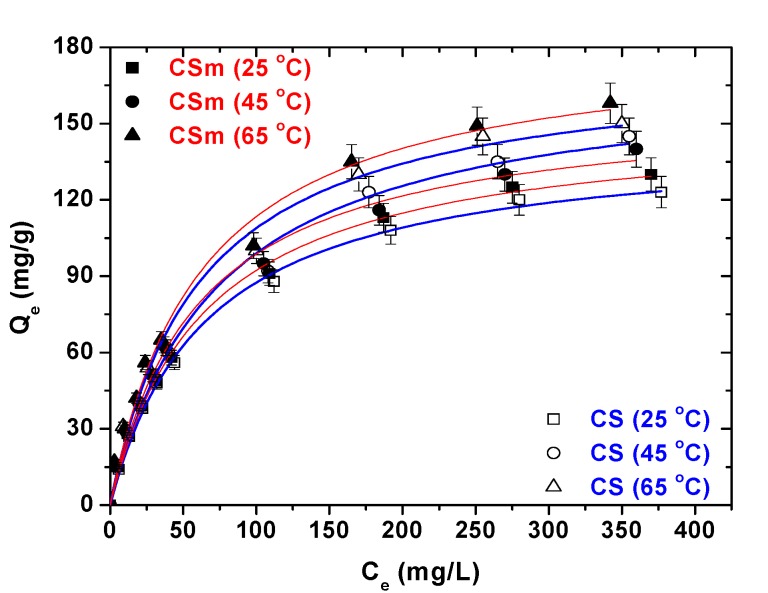
Isotherms for the adsorption of Hg(II) onto CS and CSm at 25, 45 and 65 °C (fitting to Langmuir and Freundlich equations).

Figure 10 presents the isotherms resulted from the adsorption of Hg(II) onto CS and CSm. Furthermore, Table 3 reports the maximum adsorption capacities (Q_max_) and the other isothermal parameters resulted from the fitting. The correlation coefficients (R^2^ > 0.989), which is an indication of the successful fitting, confirm that the Langmuir model results in closer prediction of the isotherm to the experimental data. The calculated maximum adsorption capacities (Q_max_) for Hg(II) removal at 25 °C (pH = 5) was 145 and 152 mg/g for CS and CSm, respectively. The latter confirms the similar adsorption behavior of two adsorbents.

**Table 3:** Equilibrium parameters for the adsorption of Hg(II) onto CS and CSm at 25, 45 and 65 °C.

Adsorbent	Langmuir equation				Freundlich equation		
	T (°C)	Q_max_ (mg/g)	K_L_ (L/mg)	R^2^	K_F_ (mg^(n−1)/n^ L^1/n^ g^−1^)	n	R^2^
CS	25	145	0.015	0.998	10.95	2.37	0.977
	45	171	0.014	0.992	10.93	2.22	0.976
	65	175	0.017	0.989	13.86	2.89	0.976
CSm	25	152	0.016	0.996	11.57	2.37	0.972
	45	158	0.017	0.994	12.54	2.39	0.988
	65	184	0.017	0.991	13.72	2.32	0.976

The effect of temperature on equilibrium is also presented in Figure 10. Increasing the temperature from 25 to 65 °C, an increase of the Q_max_ is observed. In particular, CS augmented its Q_max_ from 145 mg/g at 25 °C to 171 mg/g at 45 °C (increase of 18%) and finally 175 mg/g at 65 °C (increase of 2%). Similarly, the magnetic derivative (CSm) improved its Q_max_ from 152 mg/g at 25 °C to 158 mg/g at 45 °C (increase of 4%) and finally 184 mg/g at 65 °C (increase of 16%). However, an interesting finding of the above is the different–type change of Q_max_ of two materials during the increase of temperature adsorption. Although, CS increased its Q_max_ 18% from 25 to 45 °C, a nearly zero change was observed from 45 to 65 °C (2%). In the case of CSm, the reverse fact was observed: CSm slightly increased its Q_max_ from 25 to 45 °C (4%), while a more intense increase was for the temperatures from 45 to 65 °C (16%). The difference of the temperature–behavior of materials could be explained due to their difference modification and the existence of magnetism on CSm. The latter can behave in different manner for various temperatures. In general, as the majority of the chitosan–based materials [4], the Q_max_ augmentation with temperature increase is caused by the enhancement of the number of adsorption sites; the latter is due to the weakening and/or breaking of many structural bonds of network, existed near the edge of the active surface sites of materials. Another, possible explanation is the increase of diffusion (mainly surface diffusion [50]), which helps the penetration of mercury ions inside the structural network of materials. Also, some works revealed the role of the formation of new active adsorption sites at high temperatures [51].

Based on the equilibrium data of isotherms, thermodynamic parameters were calculated. The Gibbs free energy change, ΔG^0^ (kJ/mol), of the adsorption process is related to the equilibrium constant (K_c_) by the Van’t Hoff equation (where R is the universal gas constant and is equal to 8.314 J/mol K) [52]:


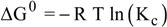
(2)

The constant K_c_ can be calculated as K_c_ = C_s_/C_e_ (where C_s_ (mg/L) is the amount adsorbed on solid at equilibrium). In addition, ΔG^0^ is related to the change in entropy (ΔS^0^, kJ/mol K) and the heat of adsorption (ΔH^0^, kJ/mol) at a constant temperature T (K), as follows:


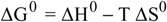
(3)

From Equations (2) and (3): 


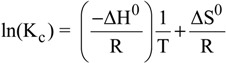
(4)

The values of ΔH^0^ and ΔS^0^ were calculated from the slope and intercept of the plot between ln(K_c_) *versus* (1/T).

The above thermodynamic parameters, at selected Hg(II) concentrations and all temperatures, are given in Table 4 (pH = 5). The negative values of ΔG^0^ showed the spontaneous adsorption of mercury ions on the adsorbent. Also, the increase found in the negative value of ΔG^0^ with an increase in temperature implies that lower temperature makes the adsorption easier. The values of ΔH^0^ were found to be positive, suggesting an endothermic process, where the adsorbate species (mercury ions) have to displace more than one water molecule for their adsorption, resulting in the endothermicity of the adsorption process. On the contrary, in a case of negative ΔH^0^ values, it would be indicated the exothermic nature of the process, thereby demonstrating that the process would be stable energetically. In an exothermic process, the total energy absorbed in bond breaking would be less than the total energy released in bond making between adsorbate and adsorbent, resulting in the release of extra energy in the form of heat. The magnitude of ΔH^0^ may also give an idea (but not sure) about the type of adsorption. The heat evolved during physical adsorption is of the same order of magnitude as the heats of condensation, *i.e.*, 2.1–20.9 kJ/mol, while the heats of chemisorption generally falls into a range of 80–200 kJ/mol. Moreover, the values of ΔS^0^ were found to be positive. A positive value of ΔS^0^ reflects the affinity of the adsorbent towards the adsorbate species. In addition, positive value of ΔS^0^ suggests increased randomness at the solid/solution interface with some structural changes in the adsorbate and adsorbent. The adsorbed solvent molecules, which are displaced by the adsorbate species, gain more translational entropy than is lost by the adsorbate ions/molecules, thus allowing for the prevalence of randomness in the system. The positive ΔS^0^ value also corresponds to an increase in the degree of freedom of the adsorbed species.

**Table 4:** Thermodynamic parameters for the adsorption of Hg(II) onto CS and CSm.

Adsorbent	C_0_ (mg/L)	T (K)	Q_e_ (mg/g)	K_c_	ΔG^0^ (kJ/mol)	ΔH^0^ (kJ/mol)	ΔS^0^ (kJ/mol K)
CS	20	298	14.02	2.33	−2.10	+18.38	+0.068
		318	15.06	3.00	−2.90		
		338	16.980	5.67	−4.87		
	100	298	56.02	1.27	−0.60	+6.10	+0.023
		318	60.08	1.50	−1.07		
		338	62.99	1.70	−1.50		
	500	298	123.10	0.33	−0.11	+5.78	+0.010
		318	145.04	0.41	−1.44		
		338	150.03	0.43	−2.44		
CSm	20	298	15.11	3.00	−0.50	+13.27	+0.054
		318	16.02	4.00	−0.80		
		338	17.08	5.67	−3.09		
	100	298	58.12	1.38	−0.20	+6.21	+0.024
		318	61.99	1.63	−0.85		
		338	65.03	1.86	−2.52		
	500	298	130.07	0.35	−0.17	+5.70	+0.010
		318	139.89	0.39	−2.03		
		338	158.01	0.46	−2.80		

### 2.3. Desorption-Reuse

**Figure 11:**
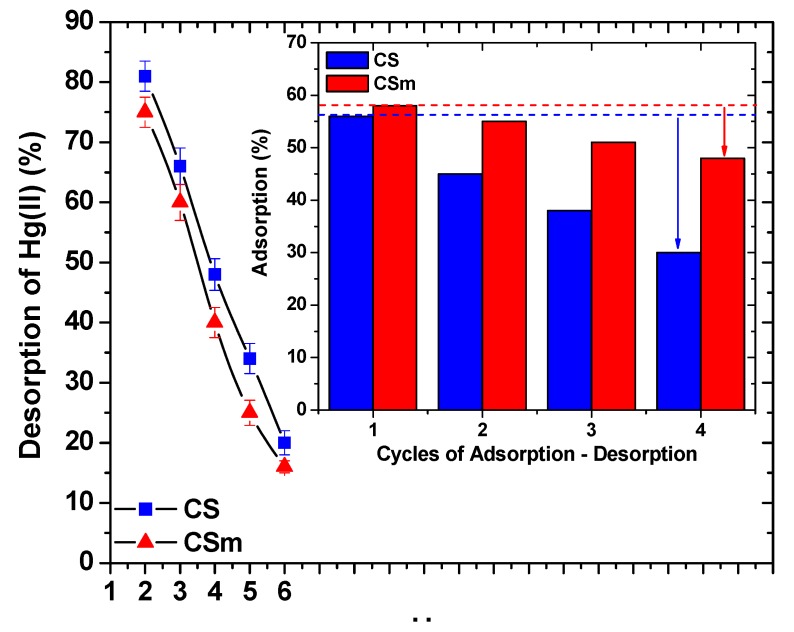
Effect of pH on the desorption of Hg(II) from CS and CSm (inset: cycles of reuse).

Firstly, desorption pH–effect experiments were carried out in order to find the optimum pH-desorption conditions. Figure 11 showed that the pH–trend of curves were different of that of adsorption. The main adsorption interaction between the chitosan derivatives prepared and Hg(II) was the chelation between mercury ions and amino groups of chitosan. However, the chelation potential is not the same both at acidic and neutral (or alkaline) pH values, given the transformation to strongly protonated amino groups at acidic media, where the chelation did not favor [4]. So, increasing the pH values, the conditions become milder and the chelation mechanism becomes stronger, affecting consequently the Hg(II) removal. However, given the adsorption process is not considered to be fully reversible, incomplete desorption can be suspected for all pH values and especially at pH = 2 (where found to be the optimum). For desorption process, the maximum desorption percentages were found to be at acidic conditions. This could be explained by the fact that the chelated bonds between Hg(II) ions and amino groups of chitosan were weakened decreasing the pH of solution, so the desorption presented higher percentages at acidic pH values [4].

The inset of Figure 11 shows the reuse potential of the materials studied via sequential cycles of adsorption–desorption. The reduction in adsorption percentages from the 1st to 4th cycle was 26% for CS (from 56% to 30%) and only 10% for CSm (from 58% to 48%). The magnetic derivative presented more stable behavior during the cycles of reuse because of its more complex network. In general, the decrease of the adsorption efficiency occurred can be attributed to several reasons as: (i) a progressive saturation of the active sites/groups of the adsorbent by mercury(II) ions, (ii) a degradation of material due to extreme pH conditions, and (iii) a progressive blocking of the active sites of adsorbents by possible impurities caused a slight decrease in the adsorption potential. However, in magnetic chitosan derivatives, the reuse ability is very high since even after the 4^th^ cycle (10% reduction), suggesting that they are promising candidates for the practical use in wastewater treatment technologies.

## 3. Materials and Methods

### 3.1. Materials

Commercial chitosan of high molecular weight was purified by extraction with acetone in a Soxhlet apparatus for 24 h. Then, its drying was carried out under vacuum at 20 °C, so the final purified chitosan product was obtained (denoted as CSp). Its average molecular weight was estimated at 3.55 × 10^5^ g/mol and the degree of deacetylation was 82 wt% [2]. The cross-linking agent used was glutaraldehyde (GLA, 50 wt% in water). Cross–linker was used, because chitosan presents high swelling degree in aqueous solutions [28]. In order to overcome this problem and given the use of chitosan (in this study) as adsorbent, it has to be cross–linked. FeCl_2_·4H_2_O (puriss. pa. > 99.0%) and FeCl_3_·6H_2_O (puriss. pa. > 99.0%) were used in the synthesis of magnetic nanoparticles. For the preparation of stock aqueous mercury(II) solutions, Hg(NO_3_)_2_·H_2_O was used (puriss. pa. ≥98.5%). All reagents were purchased from Sigma–Aldrich (Munich, Germany), while all solvents used were of analytical grade.

### 3.2. Synthesis of Adsorbents

The synthesis of chitosan derivatives was in accordance with previously published works of our research team [20,29]. The particle size of the adsorbents prepared was in the range of 75–125 μm (after grounding and sieving).

#### 3.2.1. Synthesis of Cross–linked Chitosan (CS)

2 g of CSp was dissolved in 400 mL of acetic solution (2% v/v). Then, 15 mL of GLA [approximately 2:1 aldehyde groups (–CHO) of GLA per initial amino group (–NH_2_) of chitosan] were added into the reaction flask to mix with the solution and was vigorously stirred at 25 °C for 3 h. The precipitate was washed with ethanol and distilled water in turn and dried in a vacuum oven at 45 °C. The obtained product was the cross–linked derivative of chitosan (CS).

#### 3.2.2. Synthesis of Magnetic Cross–linked Chitosan (CSm)

Initially, the preparation of magnetic nanoparticles was carried out mixing FeCl_2_·4H_2_O (3.5 g), FeCl_3_·6H_2_O (9.5 g) and double distilled water (400 mL) and stirring in a water bath at 60 °C under nitrogen for 1 h. Ammonia solution was added dropwise, purged with nitrogen, until pH = 10. The precipitate obtained was decanted in a dialysis tubing cellulose membrane (Sigma Co.) and the latter was placed in a bath filled with distilled water. The chloride ions presented in the initial suspension were slowly removed by osmosis through the membrane. The existence of Cl^–^ ions in the water bath was tested with a solution of AgNO_3_ (0.1 M). The water of the bath was replaced several times, until no more chloride ions were detectable in it. The resulting cake on the membrane surface after decanting was freeze–dried in a bench freeze drier (Christ Alpha 1–4, Osterode am Harz, Germany).

CSp (2 g) was dissolved in acetic acid solution (400 mL, 2% v/v). The prepared magnetic nanoparticles (0.75 g) were added to the above chitosan solution and the mixture was sonicated for 30 min. Then, GLA was added to mixture solution in order to cross–link chitosan. Thus, GLA (15 mL) [like for CS, the ratio was 2:1 aldehyde groups (–CHO) of GLA per initial amino group (–NH_2_) of chitosan] were added into reaction flask to mix with the solution and was vigorously stirred at 60 °C for 2 h. The precipitate was washed with ethanol and distilled water in turn and dried in a vacuum oven at 50 °C. The obtained product was the magnetic cross–linked chitosan derivative (CSm).

### 3.3. Experimental Procedure

#### 3.3.1. Adsorption-Desorption experiments

The effect of pH on adsorption was conducted by mixing adsorbent (0.02 g) with metal aqueous solution (20 mL, C_0,Hg(II)_ = 100 mg/L). The adjustment of pH can be characterized as very critical, given the variable nature of this parameter. For this reason, the adjustment of pH was done with micro-additions of HNO_3_ or NaOH. The concentrations of acid and base used were low (0.01 M) in order to have even more accuracy of pH adjustment (±0.03) not only at extremely acidic conditions (pH = 2) but also at low–acidic ones (pH = 5). The suspension was shaken for 24 h (N = 160 rpm) into a water bath to control the temperature at 25 °C (Julabo SW–21C, Seelbach, Germany). The optimum pH selected was 5.

Kinetic experiments were performed by mixing adsorbent (0.02 g) with aqueous metal solution (20 mL, C_0,Hg(II)_ = 100 mg/L). The suspensions were shaken for 24 h at pH = 5 in water bath at 25 °C (N = 160 rpm). Samples were collected at fixed–time intervals (from 5 min to 24 h). Pseudo–first (Equation (5)) [45], pseudo–second order (Equation (6)) [46] and Elovich (Equation (7)) [47] equations were used to fit the kinetic experimental data:

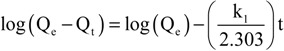
(5)

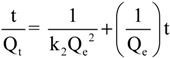
(6)

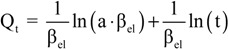
(7)
where k_1_ (min–1) and k_2_ (g mg^–1^ min^–1^) are the rate constants for the pseudo–first and –second order kinetic equations, respectively; a (mg g^–1^ min^–1^) is the initial adsorption rate and β_el_ (g/mg) is the desorption constant during any experiment exported from the Elovich equation.

The effect of temperature on adsorption was determined by mixing adsorbent (0.02 g) with of aqueous metal solutions of different initial concentrations (20 mL, C_0,Hg(II)_ = 0–500 mg/L). The suspensions were shaken for 24 h at pH = 5 in water bath at 25, 45, 65 °C (N = 160 rpm). The resulted equilibrium data were fitted to the Langmuir model [Equation (8)] [48] and the Freundlich equation (Equation (9)) [49]:

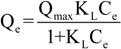
(8)

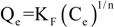
(9)
where Q_e_ (mg/g) is the equilibrium metal concentration in the solid phase; Q_max_ (mg/g) is the maximum amount of adsorption; K_L_ (L/mg) is the Langmuir adsorption equilibrium constant; K_F_ (mg^1–1/n^ L^1/n^/g) is the Freundlich constant representing the adsorption capacity; n (dimensionless) is the constant depicting the adsorption intensity.

The amount of total metal uptake at equilibrium Q_e_ (mg/g) was calculated using the mass balance equation:

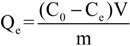
(10)
where m (g) is the mass of adsorbent; V (L) the volume of adsorbate; C_0_ and C_e_ (mg/L) are the initial and equilibrium metal concentrations in the liquid phase, respectively.

The desorption of Hg(II) from synthesized chitosan adsorbents was also investigated. Before desorption, adsorbents have to be loaded in an adsorption step. In this stage (adsorption), adsorbent (0.02 g) was added in conical flasks with 100 mg/L Hg(II) (20 mL) at pH = 5. The agitation rate was 160 rpm and the contact time was 24 h at 25 °C. Afterwards, the samples were collected and filtered, using fixed pore–sized membranes. A small fraction of Hg(II) (1%–2%) and adsorbent (1%) were retained on the filter membrane; these small variations due to filtration were neglected. Desorption experiments were carried out by mixing the collected, after adsorption, amount of Hg(II)-loaded chitosan adsorbents (0.02 g) with metal aqueous solutions of 20 mL (same volume as in the adsorption step) over a pH range between 2 and 6, at 25 °C for 24 h (N = 160 rpm). The quantitative evaluation of desorption was realized using desorption percentages, calculated from the difference between the loaded amount of Hg(II) on adsorbent after adsorption and the amount of Hg(II) in solution after desorption. This experimental procedure was done to determine the optimum desorption pH value of the Hg(II)–loaded adsorbents.

After the end of each adsorption or desorption experiment series, the measurement and analysis of Hg(II) residual concentration in the liquid phase was realized. In particular, samples were collected from the supernatant and filtered in fixed pore–sized membranes (0.40 μm) purchased from Schleicher & Schuell–MicroScience (Florida, FL, USA). Hg(II) was measured using flame atomic absorption spectrophotometer (Perkin–Elmer 1100 B, Dresden, Germany) at 254 nm. The detection limit of FAAS was 300 μg/L, that is much lower to the Hg(II) residual concentrations of this study. The detection limits are based on 98% confidence level (three standard deviations). The absorbance was converted to concentration using calibration curve.

#### 3.3.2. Instrumentation-Characterization

Scanning electron microscopy (SEM) images were performed on a Zeiss Supra 55 VP instrument (Jena, Germany). The accelerating voltage was 15.00 kV and the scanning was performed *in situ* on a sample powder. EDAX analysis was done at magnification 10 K and led to the maps of elements and elemental analysis. The FTIR spectra of the samples were taken with a FTIR–2000 spectrometer (Perkin Elmer, Dresden, Germany) using KBr disks prepared by mixing 0.5% of finely ground sample in KBr. Pellet made of pure KBr was used as the reference sample for background measurements. The spectra were recorded from 4,000 to 400 cm^–1^ at a resolution of 4 cm^–1^ with 64 co–added scans. The spectra presented are baseline corrected and converted to the transmittance mode. Thermal analysis was carried out using a TA Instrument thermal analyzer (SDT) Q500 model (TA Instruments, New York, NY, USA). The instrument had the following settings: (i) heating rate of 10 K/min and (ii) flow rate of nitrogen atmosphere equal to 100 mL/min. Approximately 25 mg of sample was used for each measurement. X–ray powder diffraction (XRD) patterns were recorded on a PW1820 diffractometer model (Philips, New York, NY, USA) with a CuKα radiation for crystalline phase identification. The sample was scanned from 20° to 80°. The magnetic property was measured on a vibrating sample magnetometer (VSM) (Oxford Instruments, Oxford, UK) at room temperature.

Swelling experiments were performed at pH = 5, because at this pH values the adsorption was found to be optimum. In particular, 1 g of adsorbent was immersed in deionized water and left to be swollen for 24 h. The pH adjustment was achieved with 0.01 M HNO_3_ and/or 0.01 M NaOH. After the pH adjustment, the material was allowed to be completely swollen, until there was no further weight increase. The swollen samples were weighted and the swelling percentage (SP, %) was calculated as:

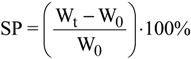
(11)
where W_t_ (g) is the weight of the swollen sample at time t, and W_0_ (g) is the initial weight of the sample before swelling.

## 4. Conclusions

The current work compares adsorptively two differently modified derivatives: (i) cross–linked with glutaraldehyde (CS), and (ii) cross–linked with glutaraldehyde and functionalized with magnetic nanoparticles (CSm). The main adsorption mechanism between amino groups of chitosan and mercury(II) ions were explained via characterization with various techniques. SEM/EDAX, FTIR, XRD, DTG, DTA, VSM, swelling tests). The optimum pH for adsorption was 5 and for desorption it was 2. CSm presented faster adsorption than CS, while its Q_max_ at 25 °C was 152 mg/g (145 mg/g for CSm). Increasing the temperature, an increase of Q_max_ was observed for both derivatives. According to thermodynamic analysis, ΔΗ^0^ > 0 suggested the endothermic nature of the process, ΔG^0^ < 0 suggested the spontaneity of the process, and ΔS^0^ > 0 showed the increased randomness at the solid/liquid interface. The reuse ability of the adsorbents prepared was confirmed with sequential cycles of adsorption–desorption. The reuse ability of CSm is very high since even after the 4^th^ cycle the reduction of the adsorption ability was 10% (26% for CS).
